# Value of ^11^C-Choline PET/CT-Based Multi-Metabolic Parameter Combination in Distinguishing Early-Stage Prostate Cancer From Benign Prostate Diseases

**DOI:** 10.3389/fonc.2020.600380

**Published:** 2021-02-01

**Authors:** Shuoming Zhou, Hongliang Fu, Changming Liu, Ziqiang Zhu, Jiabin Zhang, Wubin Weng, Jian Kang, Qiang Liu

**Affiliations:** ^1^ Department of Urology, Xinhua Hospital Affiliated to Shanghai Jiao Tong University School of Medicine, Shanghai, China; ^2^ Department of Nuclear Medicine, Xinhua Hospital Affiliated to Shanghai Jiao Tong University School of Medicine, Shanghai, China; ^3^ Department of Urology, Mindong Hospital Affiliated to Fujian Medical University, Ningde, China

**Keywords:** prostate cancer, benign prostate diseases, ^11^C-choline, positron emission tomography and computed tomography, parameter

## Abstract

**Purpose:**

The most common disadvantage of ^11^C-choline positron emission tomography and computed tomography (PET/CT) in diagnosing early-stage prostate cancer (PCa) is its poor sensitivity. In spite of many efforts, this imaging modality lacks the ideal parameter of choline metabolism for the diagnosis of PCa, and the single metabolic parameter, that is, maximal standardized uptake value (SUVmax), based on this imaging modality is insufficient. ^11^C-choline PET/CT-based multi-metabolic parameter combination can help break this limitation.

**Materials and Methods:**

Before surgery, SUVmax of choline, which is the most common metabolic parameter of ^11^C-choline PET/CT, mean standardized uptake value (SUVmean), prostate-to-muscle (P/M) ratio, metabolic tumor volume (MTV) and total lesion glycolysis (TLG) from 74 patients with histologically proven PCa were quantified. A total of 13 patients with focal chronic prostatitis without severe features and 30 patients with benign prostate hyperplasia were used for comparison. Univariable and multivariable analyses were performed to compare the patient characteristics and metabolic parameters of ^11^C-choline PET/CT. The performance of single parameters and the combination of parameters were assessed by using logistic regression models.

**Results:**

The comparable c-statistics, which mean the area under the ROC curve in the logistic regression model, of SUVmax, SUVmean, and P/M ratio are 0.657, 0.667, and 0.672, respectively. The c-statistic significantly rose to 0.793 when SUVmax and SUVmean were combined with the P/M ratio. This parameter combination performed the best for PCa cases with all biochemical recurrence risks and for PCa patients grouped by different risk. The greatest improvement over a single parameter, such as P/M ratio, was noted in the group of low-risk PCa, with values of 0.535 to 0.772 for the three-parameter combination. And in the histopathological level, the Ki-67 index is positively correlated with the P/M ratio (r=0.491, *p*=0.002).

**Conclusion:**

P/M ratio is a more ideal parameter than SUVmax as a single parameter in early-stage PCa diagnosis. According to our data, the combination of SUVmax, SUVmean, and P/M ratio as a composite parameter for diagnosis of early stage PCa improves the diagnostic accuracy of ^11^C-choline PET/CT.

## Introduction

Prostate cancer (PCa) is a common male malignant tumor worldwide with poor diagnostic accuracy of primary PCa. The treatment of PCa requires the combination of accurate diagnosis and staging with effective therapeutic methods. Generally, digital rectal examination (DRE), serum levels of prostate-specific antigen (PSA), and transrectal ultrasound (TRUS) are applied for the diagnosis of PCa. Both the tumor extension and distant metastasis were evaluated by local staging using imaging procedures, such as TRUS, Magnetic Resonance Imaging (MRI), computerized tomography (CT), and bone scintigraphy. However, there exist limitations for conventional imaging techniques like CT and MRI. For example, in a recently meta-analysis of the use of CT and MRI, a poor sensitivity of 39%–42% and specificity of 82% were shown when staging lymph nodes with even worse results in diagnosis of cancer metastasis (1). This has aroused great interest in the application of positron emission tomography (PET) which uses choline tracers for staging advanced disease.

However, the effect of PET/CT on detection of localized or locally advanced PCa within the prostate gland has been debated over the last decade (2). Previous studies have shown that the uptake values of ^11^C-choline existed a significant overlap between PCa and benign prostate hyperplasia (BPH) (3). It has also been demonstrated a high sensitivity of ^11^C-choline derivatives for locating primary PCa in the correct prostate lobe or sextant (4, 5). These studies showed that ^11^C choline PET/CT could distinguish cancer tissues from normal prostate, BPH, and localized chronic prostatitis (CP), with a low sensitivity of distinguishing benign and malignant diseases through single metabolic parameters, such as maximal standardized uptake value (SUVmax) (5), prostate-to-muscle (P/M) ratio (6), or mean standardized uptake value (SUVmean) (7).

We conducted this research to confirm the capability of ^11^C-choline PET/CT to differentiate PCa from benign prostate diseases. We also examined whether integrating the abovementioned single metabolic indexes will facilitate and further improve the diagnosis of localized or locally advanced PCa within the prostate gland, as confirmed by TRUS-guided biopsy and careful histological evaluation after surgical prostate resection.

## Materials and Methods

### Patients Enrollment

From August 2014 to January 2019, 117 unselected patients with prostate lesions and complete clinical data underwent ^11^C-choline PET/CT imaging in the Department of Nuclear Medicine, Xinhua Hospital Affiliated to Shanghai Jiao Tong University School of Medicine were enrolled in this study. All the patients understood and agreed to participate in this study and the informed consent of all involved patients could be obtained. The patients were divided into PCa (n = 74) and benign prostate disease groups (n = 43), with the latter comprising 30 cases of BPH and 13 cases of focal CP. According to the 2019 EAU/EANM/ESTRO/ESUR/SIOG Guidelines, patients newly diagnosed with PCa assessed by the risk of biochemical recurrence are divided into low-, intermediate- and high-risk groups (8). The diagnostic examination for prostate diseases included digital rectal examination, PSA and combined with TRUS. PCa diagnostic criterion was prostate biopsy or histopathology confirmed. And the histopathology of PCa group was confirmed to be adenocarcinoma. ^11^C-choline PET/CT examination, needle biopsy, and prostatectomy were all completed within one month after diagnosis. Exclusion criteria were patients who: (1) were dignosed in clinical stage M1 before operation; (2) were with status of taking anti-androgen drugs; and (3) had clinical signs of acute prostatitis.

### PET/CT Scanning

^11^CO_2_ was produced by medical cyclotron GE MINItrace II and then introduced into TraceLab FXc automatic chemical synthesis system. ^11^C-choline was synthesized by one-step method (half-life, 20 min). Blood sugar of patients were tested and the value of them were all within 7.0 mmol/L of the normal range. After fasting about 5–8 h, ^11^C-Choline PET/CT was performed. Then, the patients were intravenously injected with 7.62 ± 1.84 MBq/kg ^11^C-choline for 5 min, and PET images were obtained.

CT scanning: Contrast-enhanced CT (120 kV, 225–240 mA; 1.35:1 pitch) was acquired with 3.75 mm thickness per slice immediately before the PET acquisition, and the scanning range was from middle thigh to the top of the skull. The automatic milliampere technique was used to reduce the absorbed dose.

PET scanning: We acquired PET images from the distal margin of the pelvic floor and the acquisition time of each bed position is 3 min. Then, we used ordered-subset expectation-maximization software to reconstruct the images with CT-derived attenuation correction (matrix: 512*512). After we obtained the attenuation-corrected PET images in axial plane, CT images in coronal plane, and fused images in sagittal plane, respectively, the reconstructed PET/CT images were finally fused by Xeleris station.

### Image Interpretation

The PET images of the patients involved in this study were read independently by two experienced physicians in nuclear medicine who were unfamiliar with clinical data of the patients and imaging results before. The diagnostic criterion of primary PCa is that the prostate monofocal or multifocal ^11^C-choline uptake is significantly higher than that of periprostatic soft tissue, perirectal adipose tissue, or pelvic muscle and excludes the physiological absorption of the prostate itself (6). Given that SUV is the parameter which measures the choline metabolism of tumor foci, it cannot be used to evaluate the overall metabolism of whole tumor tissue. Thus, semi-quantitative indicators like metabolic tumor volume (MTV) and total lesion glycolysis (TLG) were introduced. Compared with the surrounding normal tissue, the choline uptake of tumor has a significantly higher MTV. The PET Volume Computed Assisted Reading (PET VCAR, GE Healthcare) software of the post-processing workstation was used to determine the threshold of drawing the edge of tumor by iterative adaptive algorithm to extract the MTV of the focus (9). The equation TLG=SUVmean×MTV(cm^3^) was used. The workstation automatically calculated the focus SUVmax, SUVmean, and prostate-to-muscle (P/M) ratio according to the region of interest (ROI), and dividing the SUV of the prostate lesion by the SUV of the psoas major muscle at the same cross-sectional level to eliminate individual differences in the physiological choline absorption in the patient, the P/M ratio was calculated.

### Histopathology

The resected prostate surface was marked with ink and then fixed with standard formalin for 24 h. Then, the prostate was continuously incised at 3–4 mm interval, from the apex of the gland to the base, perpendicular to the long axis until the incision reached the seminal vesicle junction. The sections were further fixed in formalin, embedded in paraffin, and then placed on glass slides, after which hematoxylin–eosin (H&E) was applied for staning. Other than H&E, the polyclonal rabbit anti-human Ki-67, and then goat anti-rabbit IgG (all the antibodies involved are from Abcam, Cambridge, UK) and biotinylated streptavidin–biotin immunoperoxidase conjugate were also used for staining the sections. The percentage of positive nuclei cells in more than 1,000 tumor cells in over three fields was calculated as the Ki-67 index.

Experienced pathologists (>10 years of experience) performed the histopathological examination. In line with the guidelines of the International Union against Cancer, tumor staging was carried out (10).

### Statistical Analysis

Descriptive statistics (classified variables are represented by frequency and percentage, non-normal distributed variables by median and interquartile range, and continuous random variables and the normal distribution by mean ± standard deviation) were used to describe the data. The capability of PET and non-PET imaging (pelvic CT) for diagnosing early PCa (localized and locally advanced) was compared by paired chi-square test. The difference of non-normal distributed variables was evaluated by Mann–Whitney U test. We also use the Student’s t-test to compare the differences of the mean of normally distributed continuous variables between the two groups. Metabolic parameters like MTV, TLG, SUVmax, SUVmean, and P/M ratio are continuous variables, and their correlation with PCa was studied by Spearman correlation analysis. The performance of single metabolic parameters and the combination of metabolic parameters in distinguishing PCa from control samples (BPH or CP) were tested using univariate and multivariate logistic regression models. The samples were randomly divided into the training and verification sets by stratified random sampling. The basis for layering is the risk of biochemical recurrence based on the 2019 EAU/EANM/ESTRO/ESUR/SIOG Guidelines. Finally, there were 37 patients with prostate cancer in the training set and verification set, respectively. The training set was used to fit the logistic regression model, and then we used the independent blinded verification set to test the performance of the model (11). After calculating the area under the curve (AUC) of each model, and statistically significant difference between the AUC of the parameter combination was observed by MedCalc 15.0 software (SUVmax + SUVmean + P/M ratio) and that of each single metabolic parameter. *P* < 0.05 showed a statistically significant difference, and the double-tail test was used. Kurtosis and skewness tests were used to evaluate the normality of the data. We used SPSS 24.0 statistical software to analyze all the research data.

## Results

### Metabolic Parameters of ^11^C-Choline PET/CT and Patient Characteristics

**Figure 1:**
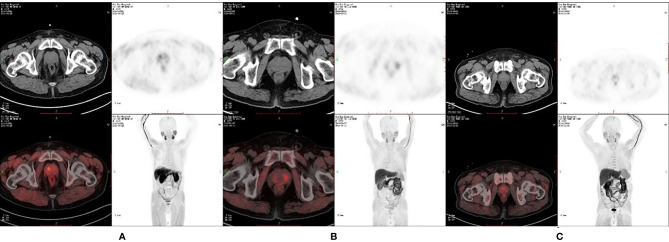
^11^C-choline positron emission tomography (PET) image of patients with different prostate diseases. **(A)** SUVmax is 5.28, SUVmean is 3.24 and P/M ratio is 8.07 in patients with PCa. **(B)** SUVmax is 3.04, SUVmean is 2.64 and P/M ratio is 3.50 in patients with benign prostate hyperplasia (BPH). **(C)** SUVmax is 2.36, SUVmean is 1.67 and P/M ratio is 2.49 in patients with chronic protatitis (CP).

**Figure 2:**
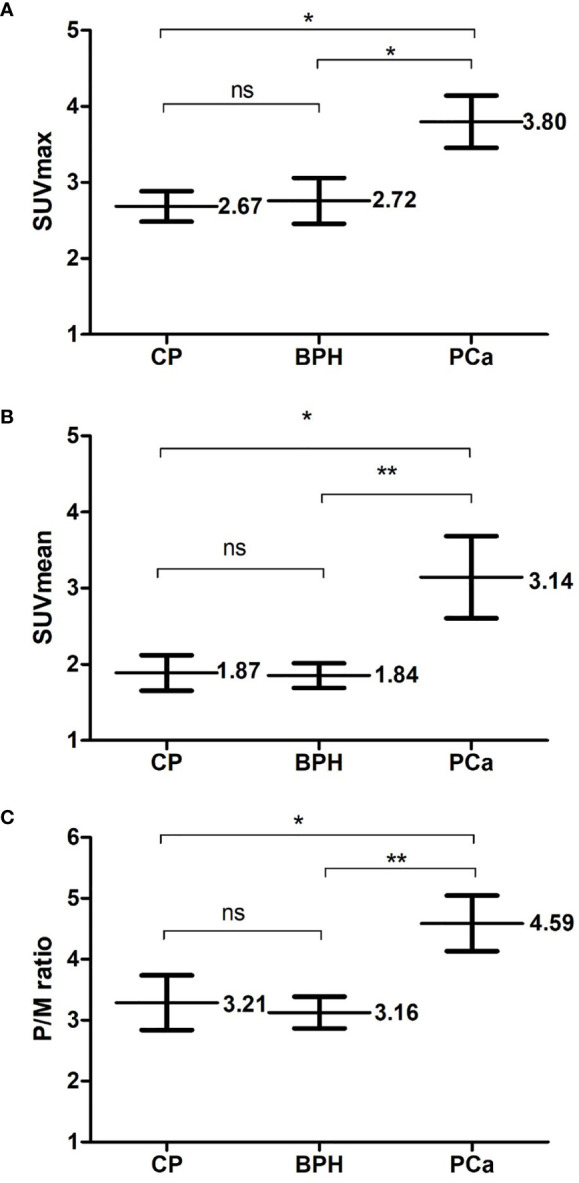
Comparison of PCa and benign prostate hyperplasia (BPH) or chronis prostatitis (CP). Three metabolic parameters of positron emission tomography and computed tomography (PET/CT): **(A)** SUVmax **(B)** SUVmean **(C)** P/M ratio were respectively compared between Pca patients and patients of benign prostate diseases. Each parameter is presented as mean ± SD. NS means no significance; *means *p* < 0.05; **means *p* < 0.01.

The final stage of analysis enrolled 117 patients undergoing ^11^C-choline PET/CT examination in our institution. Among them, 74 patients had early-stage PCa without distant metastasis, and 43 patients with benign prostate disease were initially diagnosed with suspicious lesions and finally confirmed by TRUS-guided biopsy in the control group. **Table 1** shows the demographics, clinical, and ^11^C-choline PET/CT characteristics of the patients. There was no significant differences between the PCa patients and those with benign prostate diseases in the age, body mass index (BMI), history of diabetes, and hypertension (*p* > 0.05), suggesting that the baseline data of the two groups were consistent and comparable. The PCa patients showed a significantly higher ^11^ C-choline uptakes (SUVmax, SUVmean, and P/M ratio) (*p* < 0.05) than those with benign prostate disease. The two groups showed the most significnat difference in P/M ratio (*p* = 0.008). There was no statistical differences between the two groups in terms of MTV and TLG (**Table 1**). **Figure 1** shows the SUVmax, SUVmean, and P/M ratio of patients with PCa; the values are significantly higher than those of patients with BPH or CP. In terms of the mean SUVmax level, PCa patients was 1.40 times higher than BPH patients (*p* < 0.05) and 1.42 times higher than CP patients (*p* < 0.05). The same difference can be observed in the SUVmean and P/M ratio (**Figure 2**). No statistical differences were observed between BPH and CP in terms of SUVmax, SUVmean, and P/M ratio.

**Table 1:** Clinical characteristics and ^11^C-choline positron emission tomography and computed tomography (PET/CT) metabolic parameters of patients.

	PCa (n=74)	Benign prostate disease (n=43)	*P* value
Age, median (IQR)	73(63-81)	71(64-79)	0.160
BMI, mean ± SD	23.82 ± 2.99	24.19 ± 3.29	0.600
History of diabetes, n (%)	18(24)	11(26)	0.891
History of hypertension, n (%)	26(35)	16(37)	0.915
GS PSA, (ng/ml) MTV, mean ± SD	7.51 ± 0.81 14.82 ± 5.87 9.80 ± 11.15	NA 7.85 ± 3.44 9.78 ± 5.40	NA **0.005** 0.995
TLG, mean ± SD	24.86 ± 10.36	17.21 ± 9.99	0.174
SUVmax, mean ± SD	3.80 ± 0.67	2.70 ± 0.72	**0.016**
SUVmean, mean ± SD	3.14 ± 0.74	1.86 ± 0.87	**0.010**
P/M ratio, mean ± SD	4.59 ± 0.82	3.17 ± 0.76	**0.008**

BMI, Body Mass Index; GS, Gleason Score; NA, Not Acquired; PSA, Prostate-Specific Antigen. The bold values provided means the significant meaning in statistics because their P values are less than 0.05.

### Test Characteristics of Single Parameters and Parameter Combinations

Cut-off for SUVmax, SUVmean, and P/M ratio was established through the training set to differentiate PCa from BPH or CP. A 3.277 threshold for SUVmax can detect 56.5% of PCa cases with 79.1% specificity in the verification set. At the same time, we used cut-off for SUVmean (>2.15) to discriminate between PCa and BPH or CP with 59.5% sensitivity and 74.4% specificity. In addition, when P/M ratio was used to diagnose PCa alone, the cut-off value of 3.632 was the most ideal. By combining SUVmax, SUVmean, and P/M ratio, we reached a sensitivity of 80.4% with a specificity of 86.1% in the verification data set (**Table 2**).

**Table 2:** Diagnostic ability of single parameters and parameter combination.

Parameter	Cut-off	Training set		Verification set	
		Sensitivity(%)	Specificity(%)	Sensitivity(%)	Specificity(%)
SUVmax	>3.277	57.1	77.4	56.5	79.1
SUVmean	>2.15	58.6	76.5	59.5	74.4
P/M ratio	>3.632	52.4	92	51.6	90.7
SUVmax (> 3.277) + SUVmean (> 2.15) + P/M ratio (> 3.632)					
		79.7	87.3	80.4	86.1

**Figure 3:**
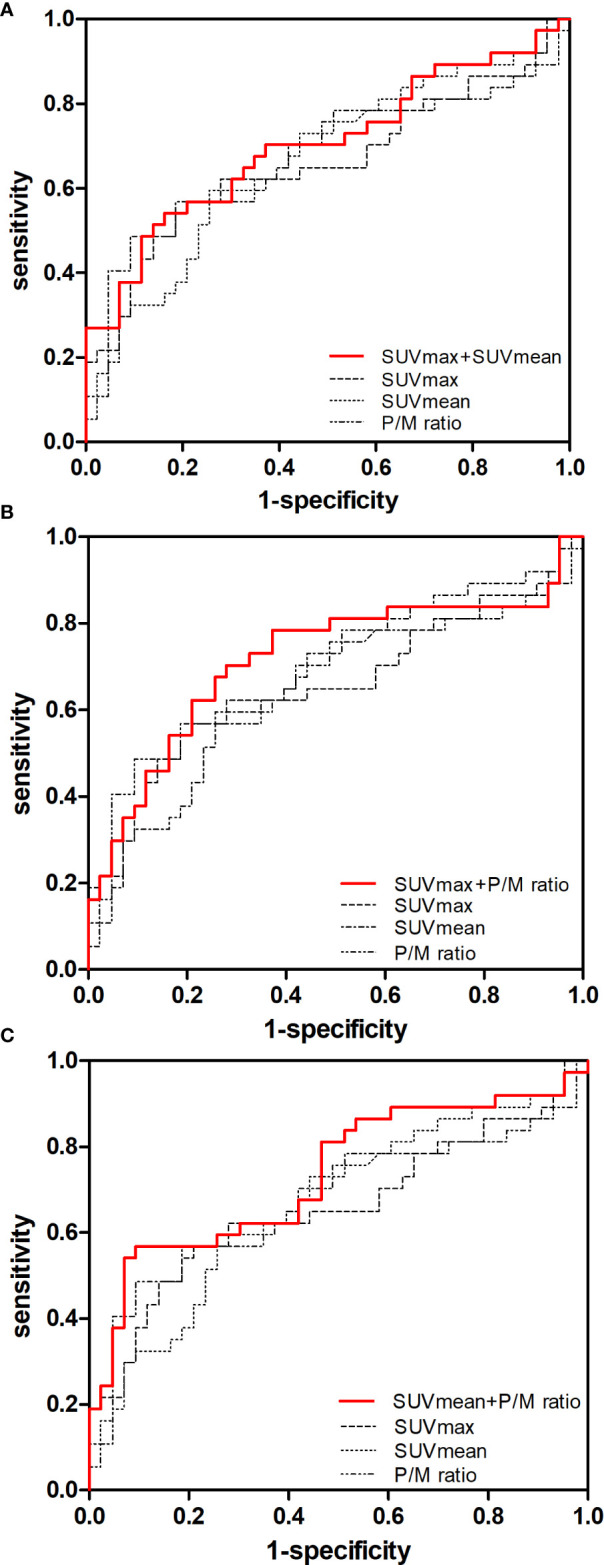
Predictive value of single parameters and two-parameter combination in the diagnosis of early-stage PCa. ROC curves for **(A)** SUVmax, SUVmean and SUVmax + SUVmean, **(B)** SUVmax, P/M ratio and SUVmax + P/M ratio, **(C)** SUVmean, P/M ratio and SUVmean + P/M ratio in controls versus patients with early-stage PCa are showed.

**Figure 4:**
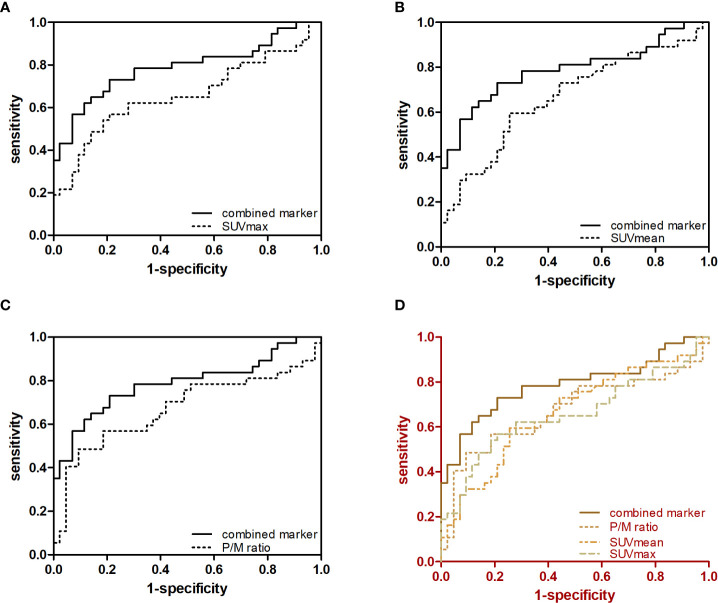
Predictive value of single parameters and three-parameter combination in the diagnosis of early-stage PCa. ROC curves for **(A)** SUVmax and SUVmax + SUVmean + P/M ratio, **(B)** SUVmean and SUVmax + SVmean + P/M ratio, **(C)** P/M ratio and SUVmax + SUVmean + P/M ratio and **(D)** SUVmax, SUVmean, P/M ratio and SUVmax + SUVmean + P/M ratio in controls versus patients with early-stage PCa are showed.

The c-statistic of SUVmax alone was 0.657 in all PCa cases in the validation set, which was close to that of SUVmean (0.667) and P/M ratio alone (0.672). Every two of the above three indicators were combined and showed no evident improvement (**Figure 3**). However, the c-statistic was significantly improved (0.793) when the SUVmax, SUVmean, and P/M ratio were combined. This parameter combination performed well for all stages and in the separation of PCa patients by biochemical recurrence risk (8). The c-statistics of low-, intermediate-, and high-risk patients with PCa were 0.772, 0.692, and 0.852, respectively. In the low-risk patients, the diagnostic effectiveness of the combined parameters is significantly higher than that of any single parameter (**Table 3**). **Figure 4** depicted the receiver operating characteristic (ROC) curves for single parameters and parameter combinations.

**Table 3:** Predictive value of different parameters in verification set.

	N	PCa patients	Low-risk	Intermediate-risk	High-risk
		37/37	9/37	12/37	16/37
SUVmax(>3.277)	AUC	0.657	0.674	0.539	0.726
*P* value		**0.0359**	**0.0345**	0.2971	0.0626
SUVmean(>2.15)	AUC	0.667	0.581	0.593	0.722
*P* value		**0.0323**	**0.0008**	0.4946	**0.0075**
P/M ratio(>3.632)	AUC	0.672	0.535	0.603	0.738
*P* value		**0.0452**	**0.02523**	0.3576	0.1355
Combined parameter	AUC	0.793	0.772	0.692	0.852

AUC, Area Under Curve; Combined parameter = SUVmax(>3.277)+SUVmean(>2.15)+P/M ratio(>3.632), P values are calculated for combined parameter model versus each single parameter. The bold values provided means the significant meaning in statistics because their P values are less than 0.05.

### SUVmax, SUVmean, and P/M Ratio Complement Each Other

**Figure 5:**
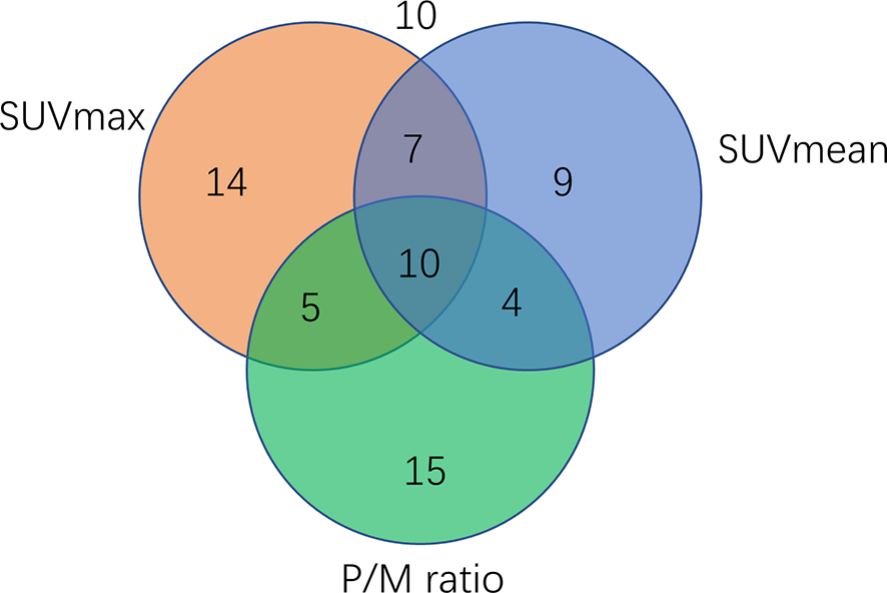
Venn diagram showing the number of PCa patients (n=74) tested positive for single parameters or parameter combination. A total of 14 samples were positive for SUVmax, nine were positive for SUVmean, 15 were positive for P/M ratio, and 10 were positive for all three parameters.

A total of 19% (12) of 74 samples were positive for SUVmax, 12% (9/74) were positive for SUVmean, and 20% (15/74) were positive for P/M ratio. A total of 6% (5/74) of the samples were positive for SUVmax and P/M ratio, another 9% (7/74) for SUVmax and P/M ratio, and 5% (5/74) for SUVmean and P/M ratio. Meanwhile, 14% (10/74) of the samples were positive for all three parameters. For all parameters analyzed, 14% (10/74) of the samples were negative. Three of these negatives were low-risk, four were intermediate-risk, and another three were high-risk PCa. **Figure 5**, shows the specific number of PCa patients whose test results are positive for single parameter and combined parameters.

### Correlation Between P/M Ratio and Ki-67 Index

**Figure 6:**
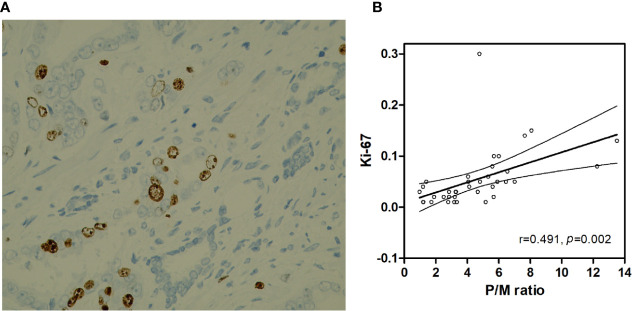
**(A)** Prostate cancer (x400 HP field), Ki-67 index is 10%. **(B)** Correlation between the Ki-67 and P/M ratio.

The Ki-67 index of 37 PCa patients in the verification set was 5.43% ± 0.92% (1%–30%). As shown in **Table 1**, the P/M ratio showed a more significant difference between the two groups than SUVmax and SUVmean (P/M ratio: *p* = 0.008; SUVmax: *p* = 0.016; SUVmean: *p* = 0.010). Thus, we analyzed the correlation between Ki-67 index and P/M ratio among the PCa patients. Pearson’s correlation analysis revealed that the Ki-67 index was positively correlated with the P/M ratio (r = 0.491, *p* = 0.002) (**Figure 6**).

## Discussion

Despite the treatment of resectable PCa has made some progress in recent years, the staging of PCa still determines the survival of PCa patients. Therefore, new strategies for diagnosing early-stage PCa are always expected, which can improve the cure rate (13). Although ^11^C-choline PET/CT guidelines were published in the past few years, the application in PCa imaging is still debated mainly because of its low sensitivity (56%–66%) in the diagnosis of primary diagnosed patients (14).

Previously, ^11^C-choline PET/CT was widely applied for screening PCa patients with the biochemical recurrence after local treatment, clinically restaging PCa, and systemically evaluating newly diagnosed high-risk patients (12, 15). Few researchers focused on the methods of improving the diagnostic accuracy of PET in low-risk PCa patients. Here, we enrolled newly dignosed patients without treatment and distant metastasis to confirm the diagnostic capability of ^11^C-choline PET/CT in the differentiation of early-stage PCa from benign prostate diseases. This study showed the meaningful role of ^11^C-choline PET/CT for diagnosing of patients with primary PCa. Sixty-four of seventy-four patients who were histologically diagnosed as PCa were successfully detected by ^11^C-choline PET/CT, while the remaining 10 patients were not.

SUVmax is thus far the most common metabolic parameter in PCa for ^11^C-choline PET/CT. SUVmax was elevated in up to 66% of patients with PCa (14) and showed a similar sensitivity of 56.5% for all PCa stages in our verification set with cut-off of 3.277. The low sensitivity of our study may be due to the inclusion of patients with BPH and CP rather than normal individuals. As the PET/CT is a powerful diagnostic tool, accompanied by the strong radiation and high examination fees, we do not recommend the PET/CT testing for the normal individuals treated as control group. And the guidelines of ethics will not permit it. Considering the relatively small sample size of this study, we used the cut-off of 3.277 for SUVmax, instead of the previously reported cut-off of 2.5 (16, 17), to minimize the influence of ethnic, geographical and other biased factors (11).

PCa has a high degree of heterogeneity, which is reflected not only in the differences in solid tumors among different patients but also in the varied metabolic states of PCa cells in the same individual (18). The heterogeneity of PCa was shown by the wide range of SUVmax measured for cancer, and some assumed ^11^C-choline PET/CT could not detect all cancers due to different metabolic states. Thus, as a single metabolic parameter based on ^11^C-choline PET/CT for non-invasive diagnosis of early-stage PCa, SUVmax alone will not suffice.

In this study, we included the five commonly used parameters of choline metabolism, namely, MTV, TLG, SUVmax, SUVmean, and P/M ratio. Univariate and multivariate analyses revealed that P/M ratio is the most ideal index, and it showed the greatest difference between the PCa and BPH or CP groups (*p* = 0.008). Cancer is mainly characterized by uncontrolled cell proliferation, and the prognosis of malignant tumors is influenced by the rate of cell division. The expression level of Ki-67 can be used to assess the proliferative activity of tumors (19, 20). And some studies have shown that Ki-67 can also reflect the function of cell metabolism, such as glucose metabolism (21). This means that the Ki-67 scores may predict the activity of choline metabolism in cells. Some reports have indicated the relationship between SUV and Ki-67 in cancers (22), but few studies have been carried out in prostate cancer. Herein, exploring the relationship between Ki-67 staining, which reflects the proliferative activity of tumor cells, and proliferation images by using ^11^C-choline PET/CT could further clarify the mechanism of ^11^C-choline uptake in prostate cancer. In this study, our data indicate that P/M ratio, an important parameter of choline metabolism, is a reliable parameter that can be used to discriminate between patients with PCa and BPH or CP. The potential value of P/M ratio as an alternative parameter for distinguishing diseases is further supported by the positive correlation of P/M ratio with Ki-67 index in the PCa patients. P/M ratio is a better promising parameter for early PCa diagnosis than SUVmax according to our research. In this condition, considering all PCa stages versus controls, the c-statistic of P/M ratio alone is 0.672, and the c-statistics are 0.535 and 0.603 when it comes to the prognostically favorable low- and intermediate-risk cases, respectively. Thus, as in SUVmax, P/M ratio alone is also inappropriate for detecting early-stage PCa. The most promising approach for the accurate diagnosis of PCa is to combine several parameters to maximize its sensitivity and specificity. According to data, the combination of SUVmax, SUVmean and P/M ratio substnatially improved the test performance. The sensitivity of this combination was increased to 80.4% and the specificity 86.1% in discriminating PCa from BPH or CP. This combination of parameters performed the best in all PCa groups with different biochemical recurrence risks and the most significant improvement was observed in low-risk PCa group. The three-parameter combination was evidently better than each single parameter (**Table 3**). PCa is characterized by multiple lesions, which are usually extremely small, and in early low-risk patients, several lesions are less than 5 mm in size (23). Given TLG=SUVmean × MTV (cm^3^), the diagnostic capabilities of TLG and MTV are reduced because they are affected by the volume of lesions. This hypothesis was supported by the fact that TLG and MTV between cancer and benign lesions under the detection of PET/CT were completely overlapped, and no cut-off value of TLG nor MTV is helpful to distinguish cancer from benign lesions.

The PCa imaging modality used worldwide is focused on the prostate-specific membrane antigen (PSMA) PET/CT (24), which plays a role in the diagnosis of PCa. However, given the difficulty of ^68^Ga/^18^F-PSMA synthesis technology and the production of the corresponding imaging equipment, ^11^C-choline PET/CT had higher popularity than PSMA PET/CT at this stage. Our research improves the diagnostic efficiency of ^11^C-choline PET/CT, which has practical significance for clinical diagnosis and treatment. These data revealed that this approach may also be of great use for the screening of patients with distant metastases. However, the selected patient cases are limited to a hospital-based population. Therefore, the feasibility of this approach requires prospective longitudinal cohort studies with more larger sample sizes of patients. In general, to our knowledge, no study has combined these three parameters to detect and localize the foci of tumors within early-stage PCa. Large sample sizes and well-designed studies are warranted to validate our findings in the future.

## Conclusion

P/M ratio is a more ideal parameter than SUVmax as a single parameter in early-stage PCa diagnosis, and its level is positively correlated with the Ki-67 index. According to our data, the accuracy of diagnosis of ^11^C-choline PET/CT was significantly improved by combining SUVmax, SUVmean, and P/M ratio as a composite parameter for diagnosing early-stage PCa, especially in the low-risk group with biochemical recurrence.

## Data Availability Statement

The original contributions presented in the study are included in the article/supplementary material. Further inquiries can be directed to the corresponding authors.

## Ethics Statement

The studies involving human participants were reviewed and approved by the Ethics Committee of Xinhua Hospital Affiliated to Shanghai Jiao Tong University School of Medicine. The patients/participants provided their written informed consent to participate in this study. Written informed consent was obtained from the individual(s) for the publication of any potentially identifiable images or data included in this article.

## Author Contributions

QL participated in the study design, cases enrollment, PET/CT acquiring, image processing, clinical management, statistical analysis, manuscript writing, and submission. SZ, JK, and HF participated in PET/CT acquiring, image processing, statistical analysis, and manuscript writing. CL participated in the study discussion, image processing, and cases enrollment. ZZ, JZ, and WW participated in cases enrollment. All authors contributed to the article and approved the submitted version.

## Conflict of Interest

The authors declare that the research was conducted in the absence of any commercial or financial relationships that could be construed as a potential conflict of interest.
